# Draft genome sequences of six multidrug-resistant *Escherichia coli* isolates from pigeons in Bangladesh

**DOI:** 10.1128/mra.01020-24

**Published:** 2024-11-14

**Authors:** Mukta Das Gupta, Mishuk Shaha, Ashutosh Das

**Affiliations:** 1 Department of Microbiology and Veterinary Public Health, Faculty of Veterinary Medicine, Chattogram Veterinary and Animal Sciences University, Chattogram, Bangladesh; 2 Department of Genetics and Animal Breeding, Faculty of Veterinary Medicine, Chattogram Veterinary and Animal Sciences University, Chattogram, Bangladesh; University of Maryland School of Medicine, Baltimore, Maryland, USA

**Keywords:** household pigeons, *Escherichia coli*, whole genome, antimicrobial resistance profile

## Abstract

Household pigeons are a potential source of multidrug-resistant (*Escherichia coli* (*E. coli*) and act as a transmission vehicle of this bacterium to humans. Here, we characterized the antimicrobial resistance profile and molecular properties of the whole genome sequences of six *E. coli* strains from household pigeons.

## ANNOUNCEMENT

Antimicrobial resistance (AMR) is a significant threat to global health. AMR genes in multidrug-resistant (MDR) *Escherichia coli* can easily be transferable to other bacteria in the gut and spread to the environment (1, 2). Human can get MDR *E. coli* from the environment and animals via the food chain (3); therefore, their presence in pigeons poses a public health risk.

Between July 2023 and October 2023, we collected cloacal swab samples from household pigeons from Chattogram and Cox’s Bazar districts of Bangladesh. These samples were then transported and investigated in the lab (23.3633 °N 91.8039°E) following a standard protocol used in a previous study (4). Briefly, swab samples in Buffer peptone water were incubated at 37°C for 24 h and then cultured overnight onto MacConkey agar at 37°C. Lactose-fermenting colonies (pink-colored colonies) on MacConkey agar were selected and subcultured on Eosin methylene blue (EMB) agar. Green metallic sheen colonies on EMB agar were identified as *E. coli*. All *E. coli* isolates were further confirmed using standard biochemical techniques described by Garcia and Isenberg (5). The Kirby-Bauer disk diffusion method (6) following the Clinical and Laboratory Standards Institute’s (CLSI) guidelines was used to test the antimicrobial susceptibility. For DNA isolation, *E. coli* isolates were cultured on blood agar for 18 h at 37°C. Subsequently, DNA was extracted using a FavorPrep Tissue Genomic DNA Extraction kit (Favorgen Biotech Corporation, Taiwan). DNA libraries were prepared using Nextera XT DNA library preparation kit (Illumina, San Diego, CA, USA). Genome sequencing was performed on an Illumina NextSeq2000 platform, which generated paired-end reads with a length of 2 × 150 bp. Reads were quality controlled using Trimmomatic v.0.39 (7) (sliding window: 4 and average quality: 20). The genome assembly was conducted using Spades v. 3.15.3 (8). The quality of genome assemblies was assessed using Quast v.5.2.0 (9). We used NCBI PGAP v.6.8 (10) for genome annotation. Phylogroup of isolates were analyzed using ClermonTyping web server (11), sequence types were predicted by MLST v.2.0.9 (12), serotype through SerotypeFinder v.2.0.1 (13), pathogenicity index by PathogenFinder v.1.1 (14), CRISPR arrays and prophages by CRISPRCasFinder web portal (15), plasmids by PlasmidFinder v.2.1 (16), antibiotic resistance genes (ARGs) by CARD v.3.2.9 (17) using web portal - RGI 6.0.3 and ResFinder v.4.5.0 (18); virulence genes by VFDB with web portal VFanalyzer (19) and VirulenceFinder v.2.0.5 (20); siderophores by antiSMASH v.7 (21); bacteriocins by BAGEL4 suite (22); and metabolic functional features by RAST v.2.0 (23). All software used default parameters unless otherwise stated.

Genomic characteristics of all the six *E. coli* strains are presented in Table 1. The *E. coli* isolates BD_EC_PG_1 to BD_EC_PG_6 contained 41, 45, 43, 41, 52, and 41 predicted ARGs, respectively, and 70, 70, 71, 71, 70, and 103 VFGs, respectively. All six *E. coli* strains were multidrug resistant, where *E. coli* strain BD_EC_PG_2 and BD_EC_PG_3 showed resistance to eight antibiotic families. The most common plasmids harbored in these isolates were Col440I, p0111, IncFIB (AP001918), and IncI1-I(Alpha). Six isolates were assigned to three sequence types-ST38, ST191, and ST1170.

**TABLE 1:** Genomic features of the sequenced six multidrug-resistant *Escherichia coli* strains isolated from household pigeons in Chattogram, Bangladesh

Isolate name	BD_EC_PG_1	BD_EC_PG_2	BD_EC_PG_3	BD_EC_PG_4	BD_EC_PG_5	BD_EC_PG_6
BioProject accession no.	PRJNA1072078	PRJNA1072078	PRJNA1072078	PRJNA1072078	PRJNA1072078	PRJNA1072078
BioSample accession no.	SAMN39727247	SAMN39727283	SAMN39727413	SAMN39727523	SAMN39727528	SAMN39727529
SRA accession no.	SRR27936543	SRR27936542	SRR27936541	SRR27936540	SRR27936545	SRR27936544
GenBank accession no.	JBGTXU000000000	JBGTXT000000000	JBGTXS000000000	JBGTXR000000000	JBGTXQ000000000	JBGTXP000000000
Total reads	11595696	11831654	9626258	10318512	11006538	12756708
Coverage (x)	333	340	276	296	317	363
Contigs	298	1585	271	422	1063	396
Contigs (>= 1000 bp)	94	150	93	88	80	97
Largest contig	467239	498746	365173	467239	524043	467239
Total length	5161147	5260190	5005064	5162360	5200676	5164464
Total length (>= 1000 bp)	5141242	5090962	4992951	5144468	5135441	5149426
N50	153838	142828	142828	153838	196266	157300
N90	36105	25515	31310	38926	44115	36518
L50	10	13	13	10	10	10
L90	37	49	43	35	32	37
GC (%)	50.66	50.56	50.62	50.67	50.4	50.67
Genes	5289	6540	5117	5318	6081	5289
Protein coding genes	4952	5660	4796	4966	5341	4952
Genome completeness	99.5%	99.55%	99.55%	99.5%	98.32%	99.5%
rRNA:	3	4	4	3	4	4
repeat_region:	2	3	2	2	2	2
tRNA:	80	80	79	80	77	84
tmRNA:	1	1	1	1	1	1
Sequence types	38	191	191	38	1170	38
Serotype	O153H9	H20	O153H9	O153H9	O1H4	O153H9
Phylogroups	D	A	A	D	B2	D
No. of Clustered Interspaced Short Repeats (CRISPR) (number of Cas clusters) (name of cas genes)	32 (8) (Cas1_0_IE, Cas2_0_IE, Cas3_0_I, Cas5_0_IE, Cas6_0_IE, Cas7_0_IE, Cse1_0_IE, Cse2_0_IE)	44(8) (Cas1_0_IE, Cas2_0_IE, Cas3_0_I, Cas5_0_IE, Cas6_0_IE, Cas7_0_IE, Cse1_0_IE, Cse2_0_IE)	44(8) (Cas1_0_IE, Cas2_0_IE, Cas3_0_I, Cas5_0_IE, Cas6_0_IE, Cas7_0_IE, Cse1_0_IE, Cse2_0_IE)	31(8) (Cas1_0_IE, Cas2_0_IE, Cas3_0_I, Cas5_0_IE, Cas6_0_IE, Cas7_0_IE, Cse1_0_IE, Cse2_0_IE)	10 (no cas gene)	31(8) (Cas1_0_IE, Cas2_0_IE, Cas3_0_I, Cas5_0_IE, Cas6_0_IE, Cas7_0_IE, Cse1_0_IE, Cse2_0_IE)
No. of prophages	6	5	5	7	7	7
Plasmids	2(Col440I, p0111)	7 (Col156, Col440I, IncFIB(AP001918), IncFIB(K), IncI1-I(Alpha), IncN, IncX1)	2 (IncFIB(AP001918), IncI1-I(Alpha)	2 (Col440I, p0111)	3 (pIGJC156, Col440I, IncFIB(AP001918)	2 (Col440I, p0111)
Resistance against antibiotic families	D-cycloserine, Piperacillin, Penicillin	D-cycloserine, Trimethoprim, Piperacillin, Penicillin, Sulfamethazine, Minocycline, Oxytetracycline Cotrimoxazole	D-cycloserine, Trimethoprim, Piperacillin, Penicillin, Sulfamethazine, Minocycline, Oxytetracycline Cotrimoxazole	D-cycloserine, Piperacillin, Penicillin	D-cycloserine, Tetracycline, Cotrimoxazole, Penicillin, Sulfamethazine	D-cycloserine, Piperacillin, Penicillin
Predicted antibiotic genes (ARGs) in CARD	41	45	43	41	52	41
Predicted virulence genes (VGs)	70	70	71	71	70	103
Predicted siderophores	Saccharide and NRPS (Non-ribosomal peptide synthetase)	Saccharide and NRPS (Non-ribosomal peptide synthetase)	Saccharide and NRPS (Non-ribosomal peptide synthetase)	Saccharide and NRPS (Non-ribosomal peptide synthetase)	Saccharide, NRP + Polyketide and RiPP:Linaridin	Saccharide and NRPS (Non-ribosomal peptide synthetase)
Predicted bacteriocins	Bottromycin, Carocin_D	Colicin_protein, Bottromycin	Colicin_protein, Bottromycin	Bottromycin, Carocin_D	Bottromycin, Colicin_Ib, Colicin-10, Colicin-E9	Bottromycin, Carocin_D
No. of subsystems (coverage, genes)	605 (64%, 3502)	399 (27%, 2005)	383 (30%, 1920)	396 (30%, 1894)	396 (29%, 1954)	393 (29%, 1892)

## Data Availability

See Table 1 for a list of accession numbers stated on.

## References

[1] Poirel L, Madec J, Lupo A, Schink A, Kieffer N, Nordmann P, Schwarz S. 2018. Antimicrobial resistance in Escherichia coli. Microbiol Spectr 6. doi:10.1128/microbiolspec.ARBA-0026-2017 PMC11633601 30003866

[2] Martínez-Vázquez AV, Vázquez-Villanueva J, Leyva-Zapata LM, Barrios-García H, Rivera G, Bocanegra-García V. 2021. Multidrug resistance of Escherichia coli strains isolated from bovine feces and carcasses in Northeast Mexico. Front Vet Sci 8:643802. doi:10.3389/fvets.2021.643802 33969038 PMC8102688

[3] da Costa PM, Loureiro L, Matos AJF. 2013. Transfer of multidrug-resistant bacteria between intermingled ecological niches: the interface between humans, animals and the environment. Int J Environ Res Public Health 10:278–294. doi:10.3390/ijerph10010278 23343983 PMC3564142

[4] Gupta MD, Sen A, Das A. 2018. Occurrence of Escherichia coli carrying Shiga toxin-producing genes in buffaloes on smallholdings in Bangladesh. Vet World 11:1454–1458. doi:10.14202/vetworld.2018.1454-1458 30532501 PMC6247868

[5] Garcia LS, Isenberg HD. 2010. Clinical microbiology procedures handbook. ASM Press, Washington, DC..

[6] Bauer AW, Kirby WM, Sherris JC, Turck M. 1966. Antibiotic susceptibility testing by a standardized single disk method. Am J Clin Pathol 45:493–496. 5325707

[7] Bolger AM, Lohse M, Usadel B. 2014. Trimmomatic: a flexible trimmer for Illumina sequence data. Bioinformatics 30:2114–2120. doi:10.1093/bioinformatics/btu170 24695404 PMC4103590

[8] Prjibelski A, Antipov D, Meleshko D, Lapidus A, Korobeynikov A. 2020. Using SPAdes de novo assembler. Curr Protoc Bioinformatics 70:e102. doi:10.1002/cpbi.102 32559359

[9] Mikheenko A, Prjibelski A, Saveliev V, Antipov D, Gurevich A. 2018. Versatile genome assembly evaluation with QUAST-LG. Bioinformatics 34:i142–i150. doi:10.1093/bioinformatics/bty266 29949969 PMC6022658

[10] Tatusova T, DiCuccio M, Badretdin A, Chetvernin V, Nawrocki EP, Zaslavsky L, Lomsadze A, Pruitt KD, Borodovsky M, Ostell J. 2016. NCBI prokaryotic genome annotation pipeline. Nucleic Acids Res 44:6614–6624. doi:10.1093/nar/gkw569 27342282 PMC5001611

[11] Beghain J, Bridier-Nahmias A, Le Nagard H, Denamur E, Clermont O. 2018. ClermonTyping: an easy-to-use and accurate in silico method for Escherichia genus strain phylotyping. Microb Genom 4:e000192. doi:10.1099/mgen.0.000192 29916797 PMC6113867

[12] Larsen MV, Cosentino S, Rasmussen S, Friis C, Hasman H, Marvig RL, Jelsbak L, Sicheritz-Pontén T, Ussery DW, Aarestrup FM, Lund O. 2012. Multilocus sequence typing of total-genome-sequenced bacteria. J Clin Microbiol 50:1355–1361. doi:10.1128/JCM.06094-11 22238442 PMC3318499

[13] Joensen KG, Tetzschner AMM, Iguchi A, Aarestrup FM, Scheutz F. 2015. Rapid and easy in silico serotyping of Escherichia coli isolates by use of whole-genome sequencing data. J Clin Microbiol 53:2410–2426. doi:10.1128/JCM.00008-15 25972421 PMC4508402

[14] Cosentino S, Voldby Larsen M, Møller Aarestrup F, Lund O. 2013. PathogenFinder - distinguishing friend from foe using bacterial whole genome sequence data. PLoS One 8:e77302. doi:10.1371/journal.pone.0077302 24204795 PMC3810466

[15] Couvin D, Bernheim A, Toffano-Nioche C, Touchon M, Michalik J, Néron B, Rocha EPC, Vergnaud G, Gautheret D, Pourcel C. 2018. CRISPRCasFinder, an update of CRISRFinder, includes a portable version, enhanced performance and integrates search for Cas proteins. Nucleic Acids Res 46:W246–W251. doi:10.1093/nar/gky425 29790974 PMC6030898

[16] Carattoli A, Zankari E, García-Fernández A, Voldby Larsen M, Lund O, Villa L, Møller Aarestrup F, Hasman H. 2014. In silico detection and typing of plasmids using PlasmidFinder and plasmid multilocus sequence typing. Antimicrob Agents Chemother 58:3895–3903. doi:10.1128/AAC.02412-14 24777092 PMC4068535

[17] Alcock BP, Raphenya AR, Lau TTY, Tsang KK, Bouchard M, Edalatmand A, Huynh W, Nguyen A-LV, Cheng AA, Liu S, et al.. 2020. CARD 2020: antibiotic resistome surveillance with the comprehensive antibiotic resistance database. Nucleic Acids Res 48:D517–D525. doi:10.1093/nar/gkz935 31665441 PMC7145624

[18] Bortolaia V, Kaas RS, Ruppe E, Roberts MC, Schwarz S, Cattoir V, Philippon A, Allesoe RL, Rebelo AR, Florensa AF, et al.. 2020. ResFinder 4.0 for predictions of phenotypes from genotypes. J Antimicrob Chemother 75:3491–3500. doi:10.1093/jac/dkaa345 32780112 PMC7662176

[19] Liu B, Zheng D, Zhou S, Chen L, Yang J. 2022. VFDB 2022: a general classification scheme for bacterial virulence factors. Nucleic Acids Res 50:D912–D917. doi:10.1093/nar/gkab1107 34850947 PMC8728188

[20] Malberg Tetzschner AM, Johnson JR, Johnston BD, Lund O, Scheutz F. 2020. In silico genotyping of Escherichia coli isolates for extraintestinal virulence genes by use of whole-genome sequencing data. J Clin Microbiol 58:e01269-20. doi:10.1128/JCM.01269-20 32669379 PMC7512150

[21] Blin K, Shaw S, Augustijn HE, Reitz ZL, Biermann F, Alanjary M, Fetter A, Terlouw BR, Metcalf WW, Helfrich EJN, van Wezel GP, Medema MH, Weber T. 2023. antiSMASH 7.0: new and improved predictions for detection, regulation, chemical structures and visualisation. Nucleic Acids Res 51:W46–W50. doi:10.1093/nar/gkad344 37140036 PMC10320115

[22] van Heel AJ, de Jong A, Song C, Viel JH, Kok J, Kuipers OP. 2018. BAGEL4: a user-friendly web server to thoroughly mine RiPPs and bacteriocins. Nucleic Acids Res 46:W278–W281. doi:10.1093/nar/gky383 29788290 PMC6030817

[23] Aziz RK, Bartels D, Best AA, DeJongh M, Disz T, Edwards RA, Formsma K, Gerdes S, Glass EM, Kubal M, et al.. 2008. The RAST server: rapid annotations using subsystems technology. BMC Genomics 9:75. doi:10.1186/1471-2164-9-75 18261238 PMC2265698

